# Clinicoradiological Features of Alexia Without Agraphia

**DOI:** 10.7759/cureus.58309

**Published:** 2024-04-15

**Authors:** Octavio Carranza-Rentería, Marc A Swerdloff

**Affiliations:** 1 Neurology, Florida Atlantic University Charles E. Schmidt College of Medicine, Boca Raton, USA; 2 Marcus Neuroscience Institute, Boca Raton Regional Hospital, Boca Raton, USA

**Keywords:** stroke, pure alexia, vascular neurology, neurology case report, neurology medical education, adult neurology, neurology, ischemic stoke

## Abstract

Alexia without agraphia is a striking vascular syndrome of the acquired inability to read words just written down. This syndrome occurs after lesions in the splenium of the corpus callosum that disconnect the angular gyrus from the visual pathway. Most of the time, a lesion in the left occipital lobe is also present, and patients present with a visual field deficit. It is a classic neurological syndrome that is rarely seen. We present two cases of alexia without agraphia seen in our hospital the same week.

## Introduction

Alexia without agraphia is a rare classic syndrome that is learned by all neurologists but rarely seen. Our current understanding of the syndrome stems from the landmark studies of the neurologist Joseph Jules Dejerine.

By the late 19th century, neurologists and neuroanatomists believed that because reading and writing were components of language, agraphia and alexia were manifestations of aphasia. This belief was derived from the work of Paul Broca, who championed the existence of a language center responsible for motor aphasia (*aphasie motrice*) [1].

A minority believed that writing and speaking were in a single language center. Others observed that aphasia did not always present with agraphia or alexia. They proposed an additional center, separate but near Broca’s area.

Dejerine, like his mentor Jean-Martin Charcot, believed alexia and agraphia were distinct phenomena from aphasia. He based his hypothesis on a case in 1891 of a man who presented with complaints of a sudden inability to read a newspaper. His neurological examination showed normal production and understanding of speech, a right homonymous hemianopia, and an inability to write spontaneously or from dictation. He retained the ability to write his name. Upon his death, his autopsy revealed a rounded lesion in the left hemisphere involving the angular gyrus that penetrated the left occipital lobe. On an axial cut of the brain, there was a lesion adjacent to the left lateral ventricle, injuring the optic radiation coursing toward the left occipital lobe. He coined the terms “word blindness” or “*cecité verbale*” to describe this clinical syndrome [2].

In 1892, Dejerine published a second case of *cecité verbale* [3]. He described a 68-year-old man who suffered two consecutive strokes. The first stroke caused the inability to read without another neurological deficit. In contrast to his prior case, this patient could write spontaneously and from dictation. Years later, he had a second stroke and developed marked difficulty writing. At autopsy, there were two anatomically distinct lesions in the left hemisphere; the oldest was in the occipital lobe at the base of the cuneus, lingual, and fusiform bodies, and the newest was in the angular gyrus and the inferior parietal lobe.

Dejerine concluded that the angular gyrus was the center for storing and understanding visual images of letters. He thought that the patient’s first lesion had disrupted tracts from the visual occipital cortex terminating in the left angular gyrus. This loss of visual information from the angular gyrus caused alexia. The second stroke destroyed the angular gyrus, causing agraphia. From these observations, Dejerine proposed two different types of word blindness: the first, alexia with agraphia, is frequently accompanied by motor aphasia and corresponds to the destruction of the left angular gyrus. The second, alexia without agraphia, arises when the left angular gyrus is spared.

This 1892 report was the first description of alexia without agraphia. Some patients can present with right homonymous hemianopia (see Case 2 below) [4]. On occasion, bedside evaluation may not find this visual deficit if it manifests as a subtle dyschromatopsia (see Case 1 below). Isolated alexia without agraphia sparing vision is rare [5].

We present two cases of alexia without agraphia seen in our hospital during the same week.

## Case presentation

Case 1

A 77-year-old right-handed woman with hypertension, hyperlipidemia, and diabetes presented with difficulty reading for four days. She was able to read each letter of a word but was unable to understand the whole word or a phrase. Her writing was unaffected. On neurological examination, she had fluent speech without dysarthria and normal repetition. She was able to name objects close to her bed. Her visual acuity was normal, and her visual fields were intact for confrontation. She had no cranial nerve deficits, motor weakness, decreased sensation, limb ataxia, or gait impairment.

**Figure 1:**
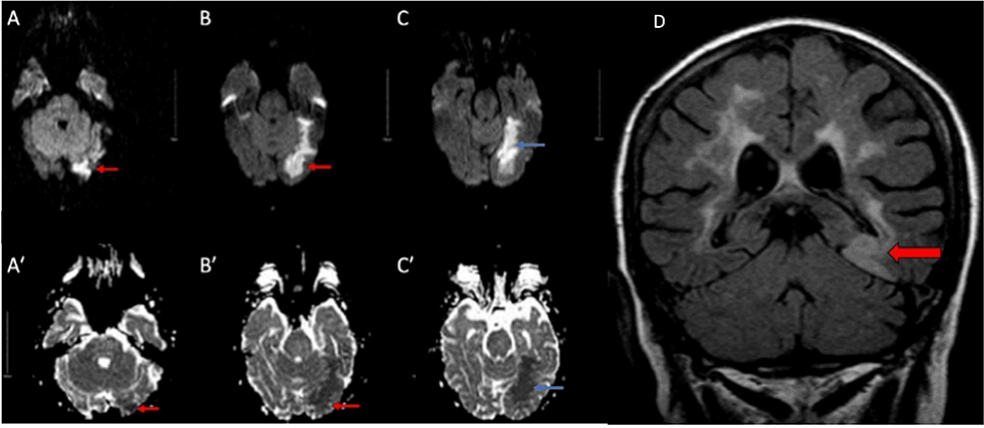
Case 1: Acute left PCA stroke (A-C’) Superior row, MRI DWI sequence. Inferior row, MRI apparent diffusion coefficient (ADC) sequence. The image shows matched DWI and ADC signals in the occipital (red arrows) and medial temporal lobes (blue arrows), consistent with acute cerebral infarction. (D) T2 FLAIR view shows ischemic lesions to the left parahippocampal gyrus and fibers adjacent to the lateral ventricle (red arrow). DWI, diffusion-weighted imaging; FLAIR, fluid-attenuated inversion recovery; PCA, posterior cerebral artery

An MRI scan of the brain showed a left posterior cerebral artery (PCA) stroke (Figure 1).

CT angiography was negative for flow-limiting vessel stenosis. The echocardiogram was negative for thrombus, patent foramen oval, or valve dysfunction. Three days of continuous cardiac monitoring failed to detect atrial fibrillation. An implantable cardiac monitor was inserted prior to discharge. She was placed on dual antiplatelet therapy (81 mg of aspirin and 75 mg of clopidogrel) for 21 days and high-dose statins (atorvastatin 80 mg) per day.

The patient was found to have subtle homonymous hemianopsia on computerized visual field testing done by neuro-ophthalmology after her hospitalization.

Case 2

**Figure 2:**
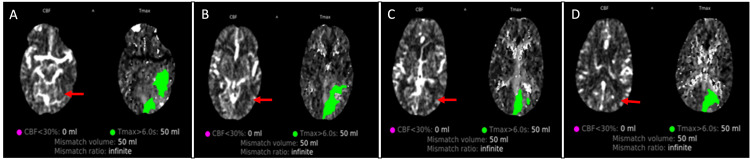
Case 2: Mismatch of CBF (ischemic core) and brain at risk (green) Advanced CT imaging shows a mismatch between normal CBF (red arrow) and perfusion (prolonged time to maximum flow (Tmax)) (green fill). The green area is the ischemic penumbra, or brain at risk. This mismatch triggered the administration of intravenous thrombolytic therapy to facilitate reperfusion. A-D are transverse sections of the brain on advanced CT imaging. CBF, cerebral blood flow

**Figure 3:**
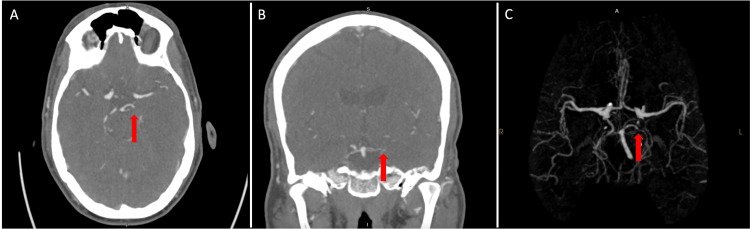
Case 2: Abrupt cutoff of blood flow in the proximal left PCA artery on CT arteriography (red arrow) (A) CT axial view. (B) CT coronal view. (C) Reconstruction of the circle of Willis. PCA, posterior cerebral artery

A right-handed 65-year-old man with hypertension presented with lightheadedness, followed by a right visual field cut. His National Institute of Health Stroke Scale (NIHSS) was 3 (two points for partial hemianopia and one point for mild expressive aphasia). A CT of the brain showed no intracranial hemorrhage. CT perfusion showed a mismatch in the left occipital region, representing a potentially reversible ischemic penumbra (Figure 2). Computed tomographic angiography of the head showed proximal occlusion of the left PCA (Figure 3). Intravenous tenecteplase was administered. The next day, right homonymous hemianopsia was found on confrontational testing. He had fluent speech with rare paraphasic errors. He had intact language comprehension and was able to name common objects. He noticed the components of the Cookie Stealing Scene in the NIHSS but was unable to grasp the concept behind the picture (Video 1).

**Video 1:** Case 2: Post-stroke Day 1 One day after the acute stroke, the patient displayed alexia without agraphia and right homonymous hemianopia.

**Figure 4:**
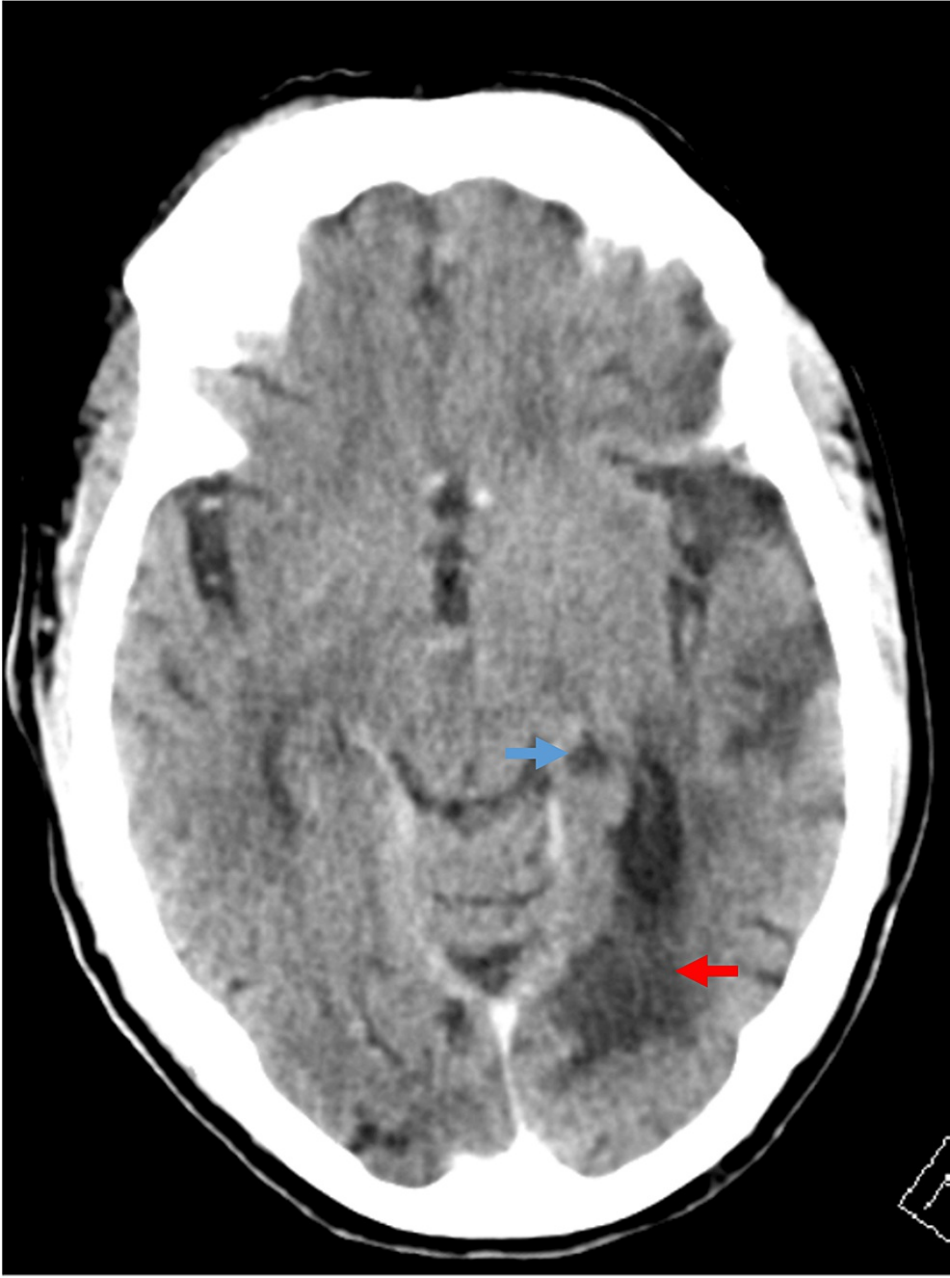
Case 2: Hypodensity in the medial temporal lobe (red arrow) and the splenium of the corpus callosum on a noncontrast CT head (blue arrow)

**Figure 5:**
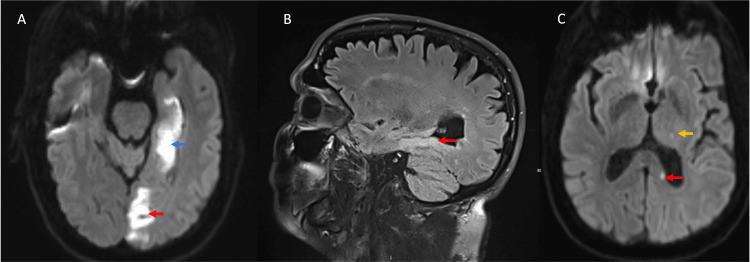
Case 2: Acute ischemic stroke in the left PCA territory MRI shows involvement of the temporal lobe (panel A, blue arrow), occipital lobe (panel A, red arrow), parahippocampal gyrus (panel B, red arrow), the thalamus (panel C, yellow arrow), and splenium of the corpus callosum (panel C, red arrow). PCA, posterior cerebral artery

Neuroimaging revealed ischemia in the left parahippocampal gyrus, left medial occipital lobe, left splenium of the corpus callosum, and pulvinar nucleus of the left thalamus (Figure 4, Figure 5). He had short-term memory loss and did not realize he had impaired reading (anosognosia). He was placed on stroke risk reduction treatment with dual antiplatelet medications (81 mg of aspirin and 75 mg of clopidogrel) and high-dose statin therapy. An implantable cardiac monitor was inserted before he was discharged to a rehabilitation facility. After three weeks, he was switched to antiplatelet monotherapy. Seven weeks after discharge, he had persistent deficits in short-term memory and an inability to read.

## Discussion

The traditional exegesis of Dejerine, and later Geschwind, is that this is a disconnection syndrome that isolates the language function of the left angular gyrus from the visual pathways [6].

Most cases of Alexia without agraphia are caused by a left PCA infarction. A left PCA territory infarct results in alexia without agraphia when visual data from the unaffected right visual cortex cannot reach the left angular gyrus. This data is interrupted at the splenium of the corpus callosum on the left and near structures, viz., the forceps major and periventricular white matter of the left lateral ventricle. This results in the disconnection of the left angular gyrus from visual information, causing alexia. When the left angular gyrus is damaged, writing is also affected (see Introduction above).

Other causes are demyelination from multiple sclerosis, migraine, seizures, infection (encephalitis or abscess), prion disease, vascular malformations, neoplasm, carbon monoxide poisoning, posterior reversible encephalopathy, eclampsia, progressive multifocal leukoencephalopathy, and COVID-19 [7-18].

Rarely, patients with alexia may present without a visual field deficit. This occurs when the pathology is located inferior to the left angular gyrus, isolating the angular gyrus from visual input from intact bilateral visual cortices.

Case 1 had a normal bedside visual field test. After discharge, formal computerized field testing showed hemi-dyschromatopsia. We point out that a subtle visual field loss may be missed if color testing is not performed.

Alexia without agraphia may also result from a lesion affecting the left lateral geniculate body of the thalamus and ipsilateral splenium. Rehabilitation of alexia includes articulation drills while reading aloud, letter-by-letter reading, and finger tracing of words. The prognosis is variable [19].

## Conclusions

Alexia without agraphia is a classic syndrome that is studied by all neurologists but is rarely seen. The syndrome was first described by Joseph Jules Dejerine in 1892. Patients are strikingly unable to read what they have just written. Most cases are secondary to a left PCA infarction. A right homonymous hemianopia is frequently seen due to the involvement of the left occipital visual cortex or lateral geniculate body.
